# The Protective Role of microRNA-200c in Alzheimer's Disease Pathologies Is Induced by Beta Amyloid-Triggered Endoplasmic Reticulum Stress

**DOI:** 10.3389/fnmol.2016.00140

**Published:** 2016-12-08

**Authors:** Qi Wu, Xiaoyang Ye, Yi Xiong, Haili Zhu, Jianting Miao, Wei Zhang, Jun Wan

**Affiliations:** ^1^Shenzhen Key Laboratory for Neuronal Structural Biology, Biomedical Research Institute, Shenzhen Peking University - The Hong Kong University of Science and Technology Medical CenterShenzhen, China; ^2^Department of Neurology, Tangdu Hospital, Fourth Military Medical UniversityXi'an City, China; ^3^Division of Life Science, The Hong Kong University of Science and TechnologyHong Kong, China

**Keywords:** Alzheimer's disease (AD), beta amyloid peptide (Aβ), microRNA, miR-200c, PTEN, endoplasmic reticulum stress (ER stress)

## Abstract

MicroRNAs are small non-coding RNAs that repress the expression of their target proteins. The roles of microRNAs in the development of Alzheimer's disease (AD) are not clear. In this study we show that miR-200c represses the expression of PTEN protein. PTEN downregulation by miR-200c supports the survival and differentiation of cultured neurons. AD is a progressive neurodegenerative disease signified by beta amyloid (Aβ) peptide aggregation and deposition. In a mouse model of AD that is induced by *APPswe* and *PS1*Δ*E9* double transgenes, we found Aβ deposition results in neuronal ER stress that induces miR200c. Pharmacological blockade of ER stress inhibited Aβ-induced miR-200c overexpression in AD brains. MiR-200c was detected in the serum of both AD mice and human AD patients. These findings suggest that miR-200c functions as part of the neuronal cell-intrinsic adaptive machinery, and supports neuronal survival and differentiation in response to Aβ induced ER-stress by downregulating PTEN.

## Introduction

Alzheimer's disease (AD) is one of most common neurodegenerative diseases affecting about 35 million people around the world (Querfurth and LaFerla, 2010), accounting for up to 70% of total dementia cases. Even though AD was first reported by Dr. Alois Alzheimer more than a century ago, the precise etiology is unknown and no effective treatments are available currently. It is well characterized that deposition of beta amyloid peptide (Aβ) and formation of intracellular neurofibrillary tangles (NFT) are predominant pathological features of AD brains (Vinters, 2015). One potential mechanism of Aβ deposition is hyper-activity in neuronal protein translation, which may eventually lead to endoplasmic reticulum (ER) overload (Fonseca et al., 2013). ER stress is believed to be the initial driver for neuronal cell loss in AD. A plethora of mechanisms such as ROS production and mitochondria dysfunction are postulated to be responsible for ER stress-mediated apoptosis in response to Aβ peptide (Cornejo and Hetz, 2013). However, how Aβ-induced ER stress leads to neuronal dysfunction still remains unknown.

MicroRNAs (miRs) are double-stranded RNAs approximately 22 nucleotides in length that play powerful regulatory roles in protein expression via mRNA decay or translational repression (Iwakawa and Tomari, 2015). Protein translation in neurons is tightly regulated by miRs. Since numerous key proteins have been identified as targets of miRs, it is well established that miRs are the most important fine-tuning regulators for many cellular process such as cell proliferation, differentiation, migration, apoptosis and senescence. Several miR families affecting Aβ deposition or tau phosphorylation are dysregulated in AD animal models or patients (Vilardo et al., 2010; Banzhaf-Strathmann et al., 2014). Recently, miRs were found to be regulated by ER stress conditions and involved in many cellular processes including ER stress-related cell apoptosis (Urra et al., 2013; Malhi, 2014; Nolan et al., 2016). ER stress might regulate miRs expression by different transcriptional factors such as XBP1 or ATF4 (Groenendyk et al., 2014; Nolan et al., 2016). However, the exact role of ER stress-miRs pathway on the pathogenesis of AD remains unclear.

By genome-wide analysis of microRNA signature in the APPswe/PS1ΔE9 double transgenic mice that harbor overexpressed Aβ peptide in the brain, we previously identified a series of miRs whose expressions were dysregulated during AD development (Luo et al., 2014). Among them, miR-200a/b/c, miR-141, and miR-429 in the miR-200 family were significantly upregulated in early age AD in mice. In nerve system, miR-200 family is enriched in olfactory and has been implicated in neuronal proliferation and differentiation (Choi et al., 2008; Pandey et al., 2015). Phosphatase and tensin homolog deleted on chromosome 10 (PTEN), a famous tumor suppressor, was found to play important roles in neurogenesis, neurite outgrowth and synaptic plasticity (Zhou and Parada, 2012). Recently, Knafo et al. discovered that PTEN is a key molecule in AD-associated post-synaptic dysfunctions (Knafo et al., 2016). In this study, we identified PTEN as a target of miR-200 family in neuronal cells. Among miR-200 family, miR-200c is the major microRNA that targets PTEN. Increased miR-200c expression in early stage AD is induced by ER stress. We provided evidence showing that upregulation of miR-200 family by ER stress exhibited protective roles via PTEN suppression in early phase of AD.

## Materials and methods

### Reagents

Mouse monoclonal antibodies against GAPDH and actin, rabbit polyclonal antibodies against PTEN, PERK, phosphorylated elF2 and CHOP were from Cell Signaling Technologies. β-tubulin III antibody, thapsigargin (TP) and sodium phenylbutyrate (4-PBA) were from Sigma-Aldrich. Anti-Phosphorylated PERK antibody was obtained from Santa Cruz Biotechnology. Aβ_1−42_ was purchased from China Peptide. The mimic or inhibitors of miR-200 family and siPTEN/control siRNAs were synthesized by Life Technologies with sequences shown in Table 1.

**Table 1:** **Sequences of synthesized miR mimics/inhibitors or siRNA**.

**Name of miRs/siRNAs**	**Sequence**
miR-200a	5′-UAACACUGUCUGGUAACGAUGU
miR-200b	5′-UAAUACUGCCUGGUAAUGAUGA
miR-200c	5′-UAAUACUGCCGGGUAAUGAUGGA
miR-141	5′-UAACACUGUCUGGUAAAGAUGG
miR-429	5′-UAAUACUGUCUGGUAAUGCCGU
miR-NC	5′-UAACGUGUCACGUCUCCGACUA
Anti-miR-200c	5′-UAACACUUGCCGGGUAAUGGUGUA
Anti-miR-NC	5′-UCUUGCCGGGCCCGAUCCAACGA
siCont	5′-UUCUCCGAACGUGUCACGU
siPTEN	5′-AACCCACCACAGCUAGAACUU

### Plasmid construction

2.3 kb PTEN 3′UTR was amplified by PCR from a human cDNA library. PCR amplicon was cloned into pMIR-REPORT vector between *Sac I* and *Mlu I* sites. A series of truncations containing different miR-200 family binding sites were also amplified and cloned into the *pMIR-REPORT* vector. For luciferase activity assay, we introduced mutations on each miR-200 family miR binding site by overlap PCR. The sequence of all constructs were confirmed by DNA sequencing.

### Animals

APP695 with Swedish mutation K595N/M596L and PS1ΔE9 double-transgenic mice (APPswe/PS1ΔE9) were purchased from the Model Animal Research Center of Nanjing University (Nanjing, China), and originally came from the Jackson Laboratory (Borchelt et al., 1996). Mouse brain tissues were collected as described previously (Wan et al., 2010). This study was performed in accordance with animal use protocols approved by the Committee for the Ethics of Animal Experiments, Shenzhen Peking University The Hong Kong University of Science and Technology Medical Center (SPHMC) (protocol number 2011-004). All animals were handled in accordance with the guidelines of the Committee for the Ethics of Animal Experiments, SPHMC.

### Cell cultures and transfection

PC12 cells were maintained in DMEM supplemented with 6% FBS and 6% HS in a 37°C incubator with 7.5% CO_2_. For NGF-induced differentiation, cells were treated with 100 ng/ml NGF in DMEM supplemented with 0.5% HS and 0.5% FBS (differentiation medium) for 4 days. The differentiation medium was refreshed every 2 days. Superior cervical ganglia (SCG) were dissected from P0 Sprague-Dawley rat pups. After removing connect tissues and blood vessels, isolated ganglia were incubated with 1% collagenase and 1% dispase for 60 min at 37°C. SCG cells were dissociated by trituration and seeded at a density of 1 × 10^5^ cells for each 35 mm dish. Cells were cultured at 37°C in a humidified 5% CO_2_ incubator. The medium was replaced 24 h later and later refreshed every 3 days. Primary cortical neurons were prepared from embryonic day 17.5 embryos of SD rats. The cortices were dissected, minced, and trypsinized for 20 min using 0.125% trypsin-EDTA and DNase I. Neurons were released by trituration and seeded at densities of 8 × 10^5^ per 35 mm dish for Western blotting, 0.5 × 10^5^ per coverslip (in a 35 mm dish) for immunostaining and 4 × 10^4^ each well in a 24 well plate for MTT assay. The cells were grown in Neurobasal-A medium supplemented with B27 and 2 mM GlutaMax. PC12 cells, SCG cells or primary cortical neurons were transfected with 100 nM of miR-200s or miR-200s inhibitors by lipofactamine 2000 according to the manufacturer's instruction.

### Cell viability assay

Twenty-four hours after miRs transfection, PC12 cells or cortical neurons were incubated with (3-(4,5-Dimethylthiazol-2-yl)-2,5-diphenyltetrazolium bromide (MTT) for 4 h to form insoluble purple formazan. After dissolving the formazan with 100 μL lysis buffer, absorbance at OD_570_ was measured with a microplate reader. All experiments were repeated at least three times.

### Luciferase reporter assay

293T cells were co-transfected with *pMIR-REPORT-PTEN-3*′*UTR* and miR-200 family for 24 h. Cell lysate was prepared and luciferase activities were measured using the luciferase reporter gene assay kit (Promega) according to the manufacturer's instructions. *pMIR-REPORT*-β*-gal* was co-transfected for normalization. All experiments were repeated at least three times.

### Real-time PCR

Total RNA were extracted from cultured cells using Trizol (Life Technologies). cDNA was synthesized from 100 ng of total RNA by miR-specific RT primers using a Reverse Transcription System (Promega). Quantitative PCR (qPCR) was subsequently performed in triplicate with a 1:4 dilution of cDNA using the 2 × SYBR green SuperMix (Bio-Rad) on a CFX96 Touch Real-Time PCR Detection System (Bio-Rad). Data were collected and analyzed with the Bio-Rad software using 2^−Δ*ΔCt*^ method for quantification of the relative miR expression levels. The expression levels of miRs in each sample were normalized against that of U6. The primers for RT-PCR and qPCR were synthesized by Life Technologies. The primer sequences were as following: MiR-200c RT primer: 5′ GTCGTATCCAGTGCGTGTCGTGGAGTCGGCAATTGCACTGGATACGACTCCATC 3′; miR-200c-forward primer: 5′ CGTAATACTGCCGGGTAATGAT 3′; miR-200c-reverse primer: 5′ GTGTCGTGGAGTCGGCAA 3′. All experiments were repeated at least three times.

### Western blotting

Cells were harvested in RIPA buffer (50 mM Tris-HCl [pH 8.0], 150 mM NaCl, 1% NP-40, 1% Nonidet-P40, 1% sodium deoxycholate and 0.1% SDS) with protease inhibitors cocktail (Sigma-Aldrich). For brain tissues, the hippocampus and cortex from APPswe/PSΔE9 mice or its littermates with different age were dissected and homogenized in D-PBS with 0.1% Triton-X100 and proteinase inhibitors cocktail. Homogenates were then mixed with equal volume of 2 × RIPA buffer at 4°C for 30 min to release proteins from tissues. Lysates were collected and subjected to SDS-PAGE. Gel separated proteins were then transferred to PVDF membrane. The blots were first probed with a primary antibody at 4°C overnight. After incubation with horseradish peroxidase-conjugated secondary antibody, signals from immunoblots were developed with the SuperSignal West Pico Chemiluminescent Substrate (Thermo-Scientific) to visualize protein bands. The signals were detected by X-ray films. Optical density was quantified by Quantity One (Bio-Rad).

### Fluorescence immunostaining

Cells were fixed with 4% PFA for 20 min, following by incubation in PBS containing with 0.4% Triton X100, 4% goat serum and 1% BSA. Fixed and permeabilized cells were then incubated with primary antibodies at 4°C overnight. After washing with PBS twice, cells were incubated with fluorescence-conjugated secondary antibodies at room temperature for 1 h. Stained cells were washed and mounted for confocal microscopic analysis (ZEISS LSM710).

### Human plasma samples and quantification of plasma circulating miR-200c

In order to evaluate the relationship between plasma miR-200c and AD severity, 14 patients with sporadic late-onset AD and 13 age-matched healthy controls were enrolled from Tangdu Hospital, Fourth Military Medical University and Peking University Shenzhen hospital. Subjects with probable AD met the National Institute of Neurological and Communicative Diseases and Stroke-Alzheimer's Disease and Related Disorders Association (NINCDS-ADRDA) diagnostic criteria (McKhann et al., 1984), and none reported to have a family history of dementia. Individuals with Mini-Mental State Examination (MMSE) scores of 15–26 were marked with mild AD, and those with MMSE scores of 5–14 were placed in the moderate to severe AD group. Written informed consent was obtained from all individual participants included in the study. All procedures followed were in accordance with the ethical standards of the responsible committee on human experimentation. Two milliliters of whole blood from each patient were collected in EDTA tubes followed by plasma separation by centrifugation. The plasma was kept at −80°C. Characteristics of the selected patients are summarized in Table 2.

**Table 2:** **Patient characteristics**.

**Patient no**.	**Sex**	**Age**	**AD severity**
A1	Female	61	Mild
A2	Female	65	Severe
A3	Female	57	Moderate
A4	Male	63	Moderate
A5	Male	56	Moderate
A6	Female	68	Mild
A7	Male	77	Moderate
A8	Female	63	Moderate
A9	Male	60	Moderate
A10	Female	71	Mild
A11	Male	64	Mild
A12	Male	75	Mild
A13	Male	65	Mild
A14	Male	54	Mild
N1	Male	57	—
N2	Female	59	—
N3	Male	72	—
N4	Male	80	—
N5	Male	65	—
N6	Female	61	—
N7	Male	62	—
N8	Female	60	—
N9	Male	57	—
N10	Male	58	—
N11	Male	60	—
N12	Female	75	—
N13	Female	56	—

The plasma miR-200c levels of AD patients and healthy controls were detected by TaqMan microRNA qRT-PCR assays as previously described (Wang et al., 2015). The TaqMan probe for miR-200c is 5′-FAM-CACTGGATACGACTCCATCATTACC- TAMRA-3′.

### Statistical analyses

Data are expressed as the mean ± SEM. Statistical comparisons were made between two groups with the Student's *t*-test. Differences among multiple groups were analyzed by ANOVA with *post-hoc t*-tests. *P*-value smaller than 0.05 is considered statistically significant.

## Results

### Identification of PTEN as a target of miR-200 family

**Figure 1:**
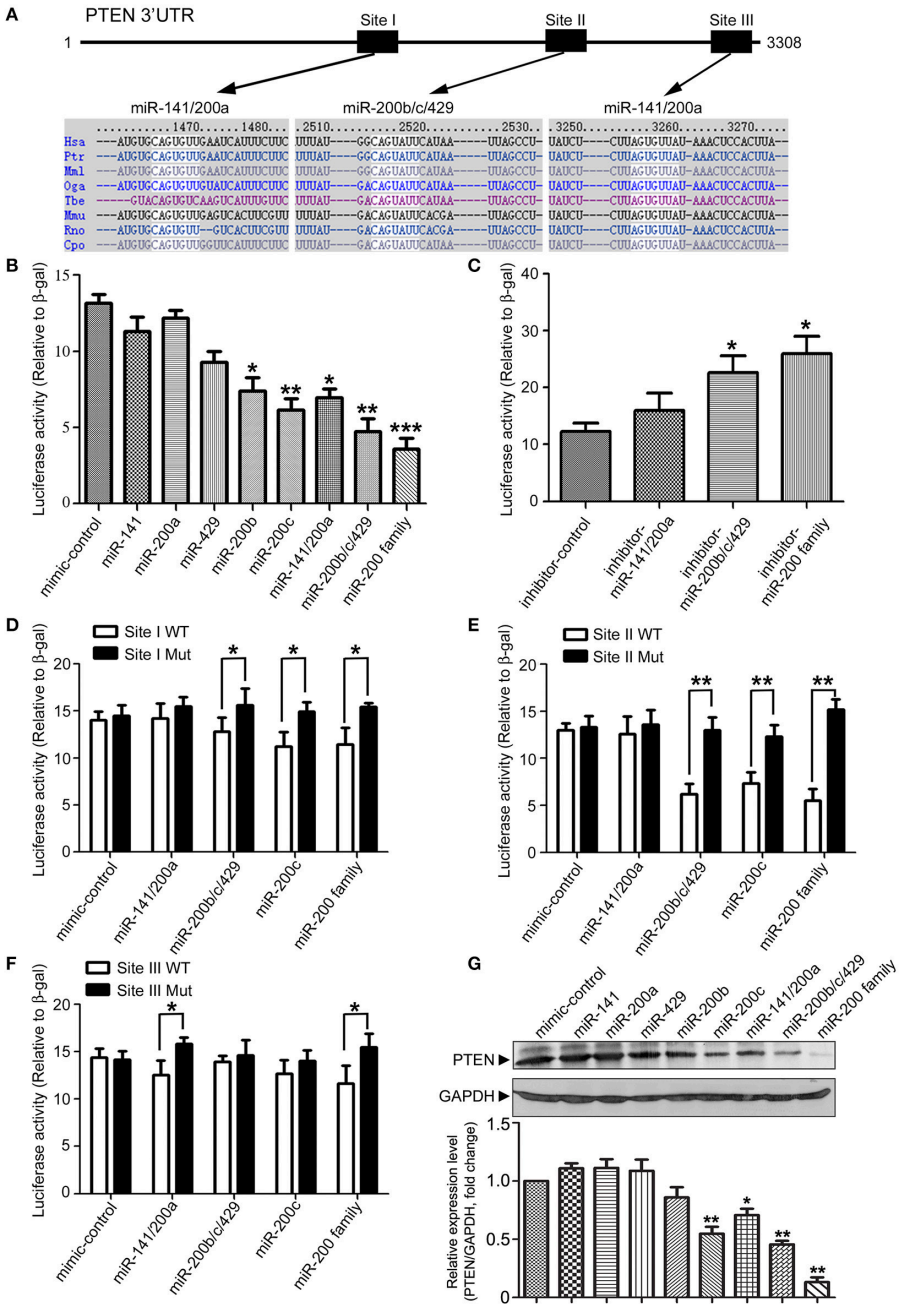
**Identification of PTEN as a target of microRNA 200 family. (A)** Schematic diagram of the putative mmu-miR-200 family binding sites in mouse PTEN 3′-UTR. As predicted by Targetscan (version 6.2, 2012), three miR-200 family bind sites (Site I and Site III for miR-141/200a, Site II for miR-200b/c/429) are highlighted. **(B)** 293T cells were transiently transfected with a *pMIR-Reporter* construct containing the full-length of 3′UTR of PTEN and an internal control plasmid (β*-gal-pCMV*), together with either individuals of miR-200 family (miR-141, miR-200a, miR-429, miR-200b, and miR-200c) or different mixtures of miR-200 family (miR-141/200a, miR-200b/c/429 or the whole miR-200 family). Luciferase activity was measured and normalized against the β-gal activity in the same samples. Experiments were repeated for three times. Data are represented as mean ± SEM. (*n* = 3) (^*^P < 0.05, ^**^P < 0.01, ^***^P < 0.001, compared to miR-mimic-control transfected cells). **(C)** *pMIR-Reporter-PTEN* was transfected into 293T cells with different combination of miR-200 inhibitors (miR-141/200a inhibitors, miR-200b/c/429 inhibitors or miR-200 family inhibitors). Luciferase activity was measured similar as described in **(B)** and three independent experiments were performed. Data are represented as mean ± SEM. (*n* = 3) (^*^P < 0.05, vs. miR-inhibitor-control transfected cells). **(D–F)** Seed sequence of miR-200 binding sites (Site I to III) in 3′UTR of PTEN were mutated individually. *pMIR-Reporter* constructs containing either wild type or mutant fragments of PTEN 3′UTR were co-transfected into 293T cells with miR-200 family miRs (miR-141/200a, miR-200b/c/429, or miR-200c). Twenty four hour after transfection, cells were collected and subjected to luciferase assay. Experiments were repeated for three times independently. Data are represented as mean ± SEM. (^*^P < 0.05, ^**^P < 0.01, compared to wild-type). **(G)** 293T cells were transfected with different miR-200 family members. Cell lysates were subjected to immunoblotting with antibodies against PTEN or GAPDH. The experiments were repeated for three times. Gray degree values are quantified by Image J software. The result was shown as the mean ± SEM. (Lower panel).

In our previous study, we identified a series of microRNAs that are differentially expressed in the brains of APPswe/PSΔE9 transgenic mice (Luo et al., 2014). Among them, microRNA-200 family showed a dynamic expression profile during AD development in the APPswe/PSΔE9 transgenic mice. In order to understand the role of miR-200 family in AD development, we employed the TargetScan (version 6.2, 2012) database to predict potential miR-200 target genes. MiR-200 family can be divided into two groups according to the seed sequences (group I: miR-141 and miR-200a; group II: miR-200b, miR-200c, and miR-429). Database prediction indicated that PTEN has three miR-200 family binding sites in its 3′UTR and may be one of the potential targets of miR-200 family (Figure 1A). To explore the potential roles of miR-200 family members in translational regulation of PTEN expression, we co-transfected a luciferase reporter construct containing PTEN 3′UTR together with different miR-200 family members. Cotransfection of miR-200b, miR-200c, a mixture of group I, group II or the whole family of miR-200s all resulted in a decrease in luciferase activity (Figure 1B). Overexpression of miR-200 family inhibitors dramatically enhanced luciferase reading (Figure 1C). To identify the binding site of miR-200 that plays the most important role in miR-200-regulated PTEN protein expression, we generated three different site-directed mutations on PTEN 3′UTR. Our results showed that miR-200b, c and 429 mainly target site I and II, whereas miR-141, 200a mainly target site III (Figures 1D–F). Among the three sites, site II seems to have the most important role in the regulation of PTEN expression by miR-200 family. Among the five miR-200 family members, miR-200c plays the most important regulatory role and was used to represent miR-200 family in the following parts of the study (Figures 1D–F). We found that miR-200c significantly suppresses the level of PTEN expression in 293T cells (Figure 1G), which is consistent with aforementioned results in the luciferase reporter assay. On the other hand, transfection of miR-200 inhibitors to HCT116 colon cancer cells that have high endogenous levels of miR-200 resulted in increased expression of PTEN (Figure S1). Taken together, the miR-200 family, especially miR-200c, directly targets the 3′UTR of PTEN and inhibits its protein expression.

### miR-200c and PTEN are involved in neuronal survival and neurite outgrowth

**Figure 2:**
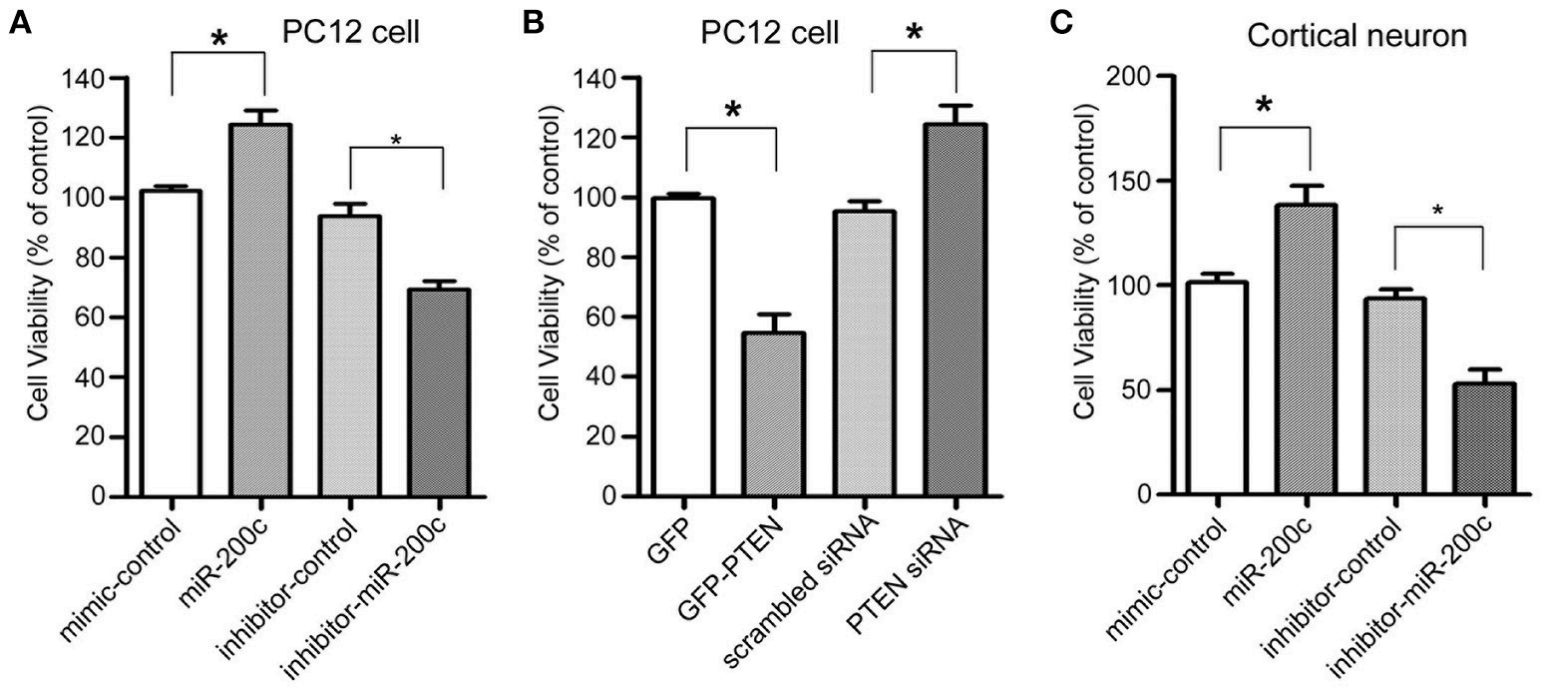
**Effects of miR-200c and PTEN on neuronal cell viability. (A)** PC12 cells were transfected with miR-200c mimic or inhibitor for 24 h, followed by NGF-induced differentiation [Cells were treated with 100 ng/ml NGF in DMEM supplemented with 0.5% HS and 0.5% FBS (differentiation medium)] for 48 h. Cell viability was measured by MTT assay. Three independent experiments were performed. Data are represented as mean ± SEM. (^*^P < 0.05, miR-200c vs. mimic/inhibitor control). **(B)** PC12 cells were transfected with GFP-PTEN or PTEN siRNA. After 2 days differentiation induced by NGF, MTT assay was performed to measure the cell viability. Three independent experiments were performed. Data are represented as mean ± SEM. (^*^P < 0.05, vs. control). **(C)** Rat primary cortical neurons were transfected at DIV0 with miR-200c mimic or inhibitor. Cell viability was measured by MTT assay at DIV3. Three independent experiments were performed. Data are represented as mean ± SEM. (^*^P < 0.05, miR-200c vs. mimic/inhibitor control).

As a key negative regulator on PI3-Kinase signaling pathway, PTEN plays an important role in regulating both neuronal survival and neurite outgrowth. We therefore investigate the role of miR-200c on neuronal survival. Overexpression of miR-200c significantly promoted cell viability of both PC12 cells and cultured rat cortical neurons, while overexpression of antisense oligo against miR-200c reduced the survival rate (Figures 2A,C). Knocking down of PTEN resulted in similar increase in the percentage of living cells compared to the overexpression of miR-200c, whereas overexpression of PTEN protein caused significant cell death (Figure 2B).

**Figure 3:**
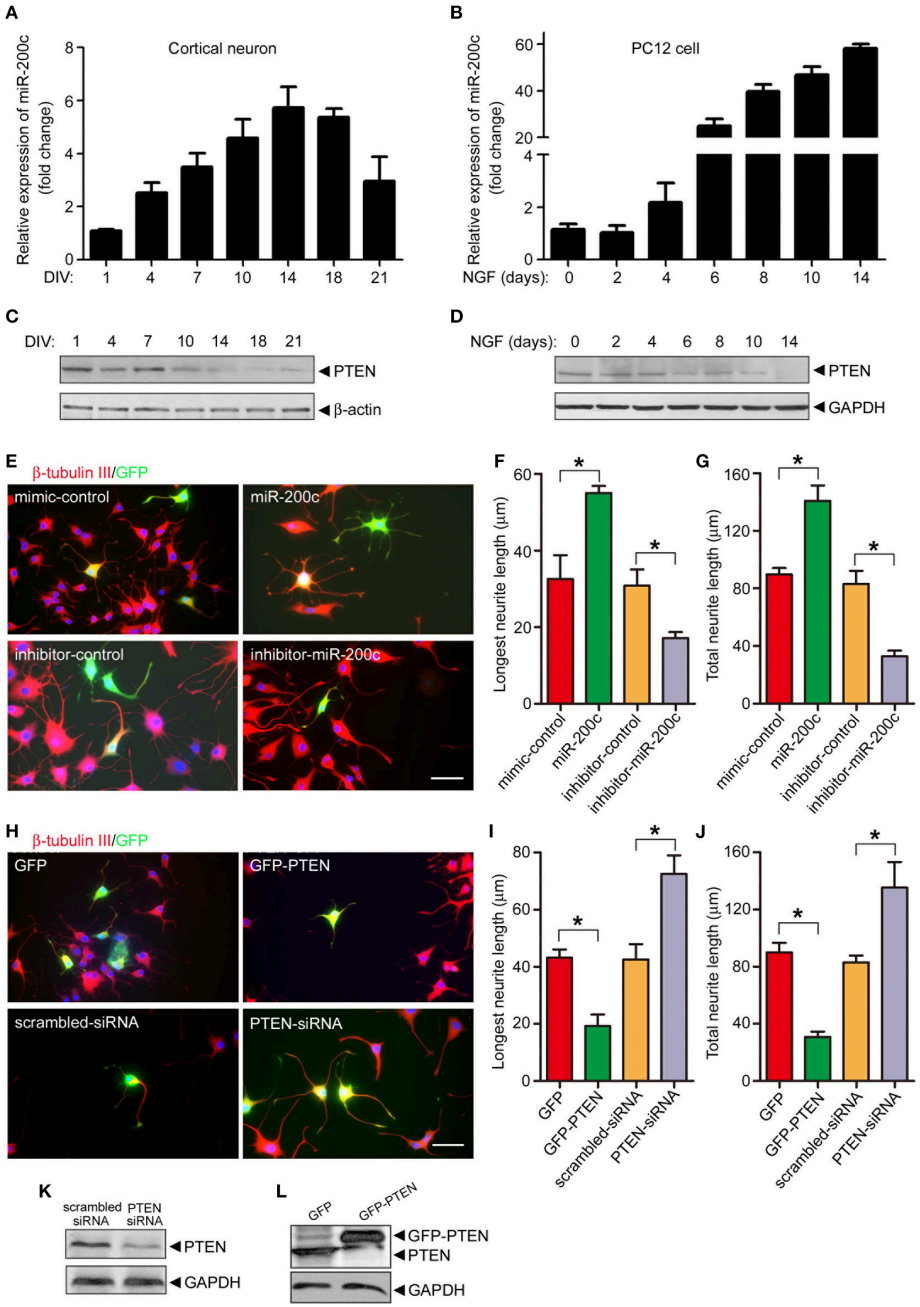
**miR-200c is important for neurite outgrowth in PC12 cells and cultural cortical neuron. (A–D)** Total RNA was extracted from rat primary cortical neurons **(A)** or NGF-treated PC12 cells **(B)** followed by qPCR to detect the expression of miR-200c. Data was shown as the fold change compared with DIV1 (cortical neuron) or before NGF treatment (PC12 cell). The lysates of primary cortical neurons **(C)** or PC12 cells **(D)** were subjected to immunoblotting analysis using PTEN antibody. **(E–G)** PC12 cells were transfected with miR-200c mimic or inhibitor together with GFP. Twenty four hours after transfection, cells were treated with NGF for another 48 h. Neurite outgrowth was visualized by immunostaining with β-tubulin-III **(E)**. Longest neurite length **(F)** and total neurite length **(G)** from 80 to 100 transfected cells were measured by the Metamorph software. Three independent experiments were performed. Data are represented as mean ± SEM. (^*^P < 0.05, miR-200c vs. mimic/inhibitor control). **(H–L)** PTEN was overexpressed or knocked-down by the transfection of GFP-PTEN or PTEN siRNA followed by NGF treatment and measurement of neurite outgrowth. Data are represented as mean ± SEM from at least 3 independent experiments. (^*^P < 0.05 vs. mimic/inhibitor control. Scale bars: 20 μm). PTEN expression level was measured by immunoblotting **(K,L)**.

In addition to neuronal cell survival, we also tested whether miR-200c regulates neurite outgrowth. Expression of miR-200c increased gradually during in cultured cortical neurons and NGF-treated PC12 cells (Figures 3A,B). The level of PTEN protein gradually decreased in the same process (Figures 3C,D), further supporting the notion that PTEN is a target of miR-200c. To further investigate the role of miR-200c on neurite outgrowth, miR-200c mimic or inhibitor was transfected into PC12 cells together with GFP as a transfection indicative marker. Neurites were stained by β-tubulin-III antibody (Figure 3E). Overexpression of miR-200c enhanced NGF-induced neurite outgrowth, whereas inhibition of miR-200c showed the opposite effect (Figures 3E–G). Similar results were obtained in SCG neurons (Figures S2A–C).

Since we have identified PTEN as a major target of miR-200c, we then altered the level of PTEN protein by overexpression or knocking down and examine neurite outgrowth by immunostaining same as above (Figure 3H). PTEN overexpression significantly reduced the length of the longest and overall neurite in both NGF-treated PC-12 cells (Figures 3H–L) and SCG neurons (Figures S2D–F). In contrast, knocking down of PTEN expression promoted neurite outgrowth in these two cells types.

**Figure 4:**
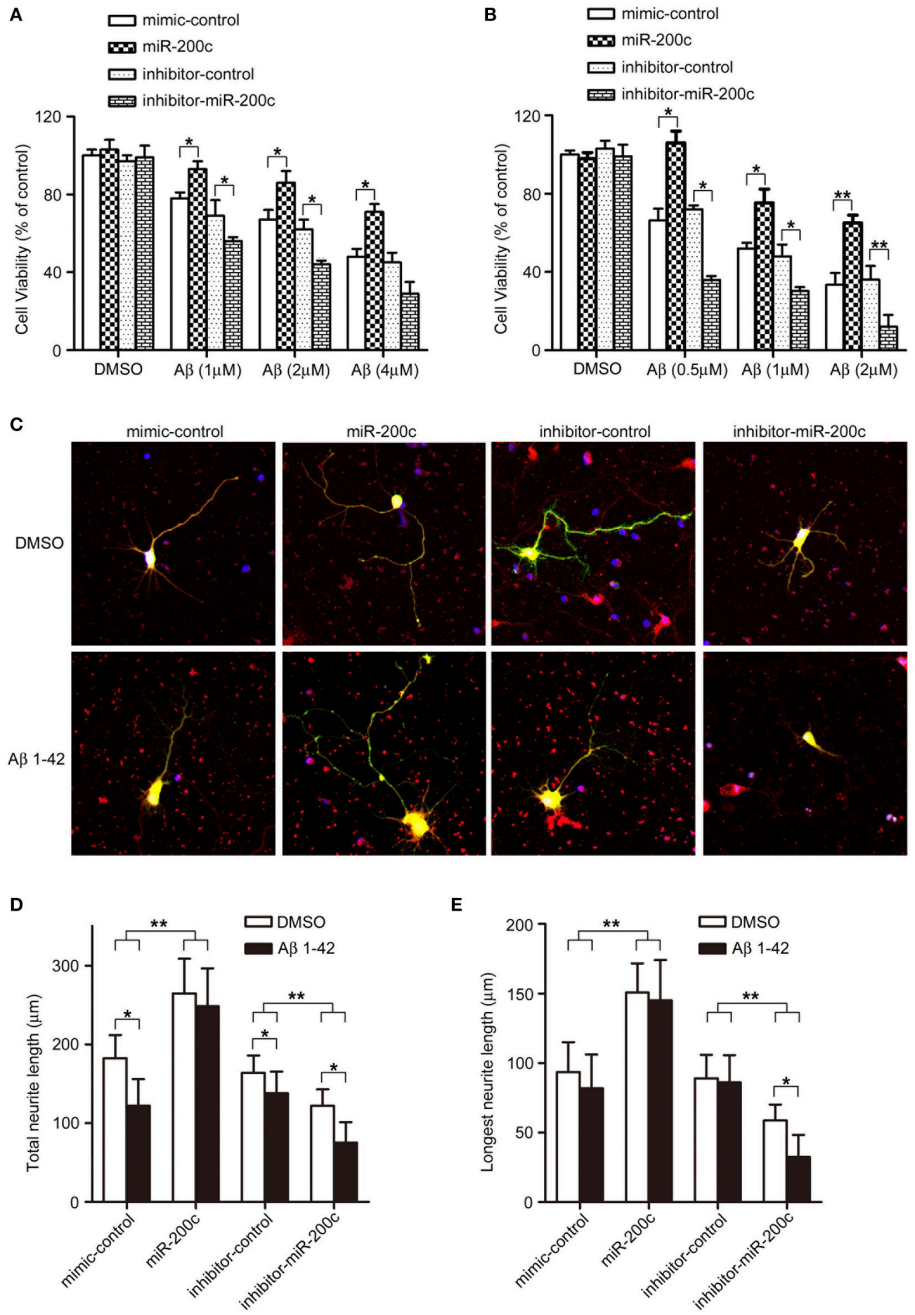
**miR-200c protects neurons from Aβ-induced damage. (A,B)** PC12 cells **(A)** or rat cortical neurons **(B)** were transfected with miR-200 (mimic or inhibitor). After 48-h culturing (PC12 cells were differentiated by NGF), the cells were treated with indicated doses of Aβ_1−42_ for 48 h. Cell viability was measured by MTT assay. Three independent experiments were performed. Data are represented as mean ± SEM. (^*^P < 0.05, ^**^P < 0.01). **(C)** Rat cortical neurons at DIV0 were transfected with miR-200c mimic or inhibitor with GFP. Twenty four hours after transfection, cells were treated with Aβ (1 μM) for another 48 h. AChE positive cells was visualized by immunostaining (Red). **(D,E)** Total **(D)** or the longest neurite length **(E)** were measured as described in Figure 3. Three independent experiments were performed. Data are represented as mean ± SEM. (^*^P < 0.05, ^**^P < 0.01).

The effects of miR-200c on neurite outgrowth were not observed in rat cortical neurons (Figure S2G), which may stem from the difference where PC12 cells and SCG neurons use TrkA signaling but cortical neurons mainly use TrkB signaling. However, TrkA signaling is present in a small portion of cortical neurons that are positive in AchE. The damage of this portion of neurons are important in cognitive impairment in AD. To test if TrkA signaling plays an important role in miR-200c mediated regulation of neurite outgrowth, AChE was stained to label those cortical neurons that are TrkA positive. As shown in Figure S2H and Figure 4, overexpression of miR-200c in AChE positive cells resulted in enhanced neurites outgrowth and knocking down of miR-200c caused a significant reduction in neurites length in AChE positive cells (Figure S2H; Figure 4C, upper panel; Figures 4D,E, white bars). These results indicate that miR-200c regulates neurite outgrowth of TrkA positive neurons. TrkA seems to be the regulatory target of miR-200c in the process of neurite outgrowth.

### miR-200c plays protective roles in Aβ-induced neuronal cell damage

Since miR-200c is dysregulated in the AD brain and is important for neuronal survival at least in part through regulating the expression of PTEN, we examined the role of miR-200c in Aβ-induced neuronal cell death. Overexpression of miR-200c reduced the insult of Aβ in both PC12 cells and cortical neurons (Figures 4A,B), indicating that the miRNA plays a protective role against Aβ-induced neuronal damage.

Neurite outgrowth is also impaired by Aβ treatment in cortical neurons. Overexpression of miR-200c rescued such reduction upon Aβ treatment, whereas transfection of miR-200c inhibitors further exacerbated neurite outgrowth inhibition (Figure 4C, lower panel; Figures 4D,E, black bars).

### miR-200c expression is correlated with neuronal ER stress

**Figure 5:**
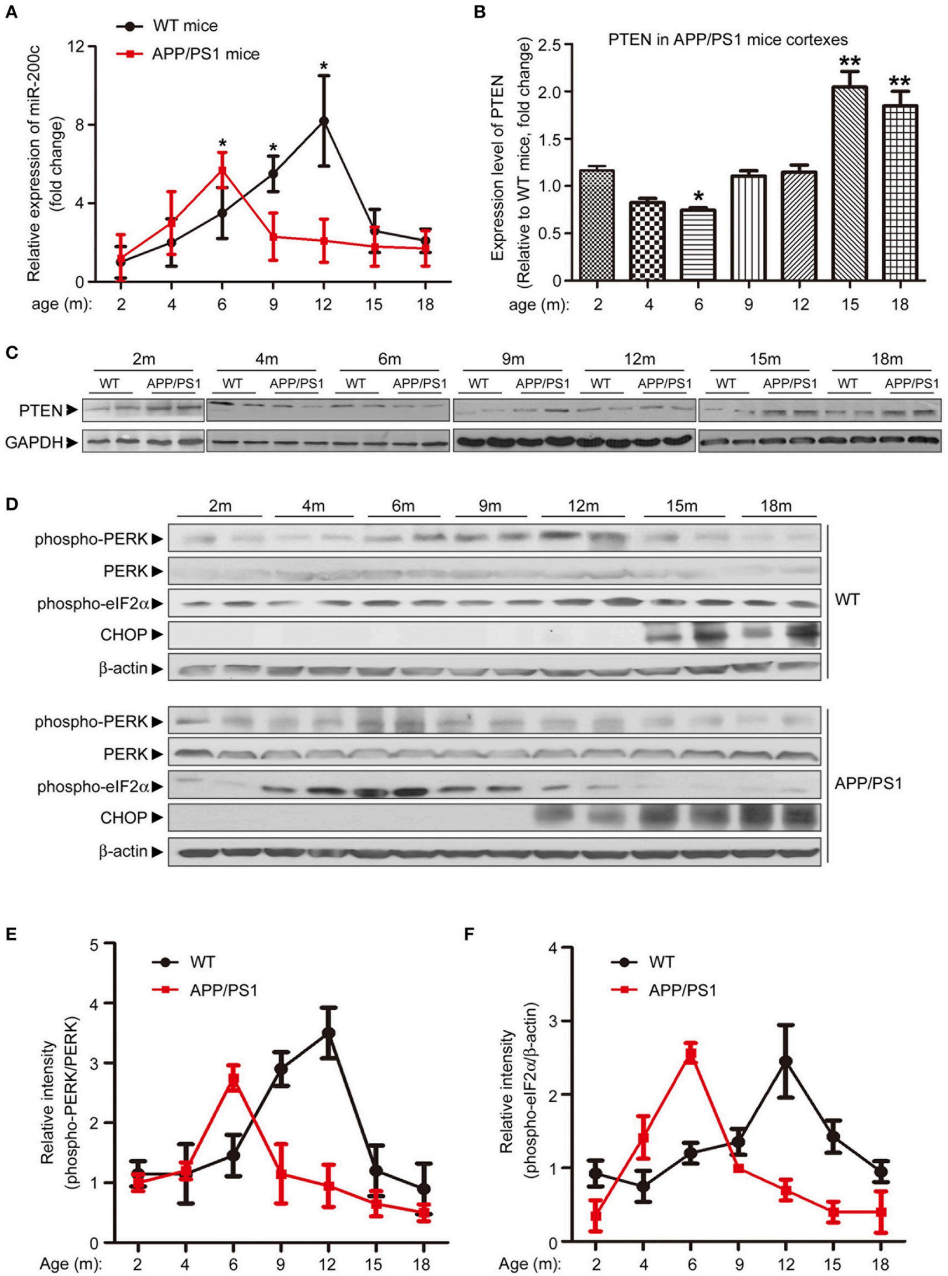
**Expression pattern of miR-200c and PTEN in APP/PS1 mice brains is correlated with ER stress makers. (A)** Expression profile of miR-200c during the different developmental stages of APP/PS1 mice cortexes were examined by analyzing six pairs of APP/PS1 mice and WT mice at each stage. Total RNA was extracted from mice cortexes. Relative expression of miR-200c was detected by qPCR Data was represented as fold changes compared with the level at 2-month old; ^*^P < 0.05). **(B,C)** PTEN expression profile during the different developmental stage of APP/PS1 mice cortexes was determined by Western blotting. Lysate extracted from APP/PS1 or WT mice cortexes were subjected to immunoblotting with PTEN antibody **(C)** and the signal was quantified (APP/PS1 vs. age-matched WT, ^*^P < 0.05, ^**^P < 0.01) **(B)**. **(D)** Cortex from APP/PS1 or WT mice brains were lysed by RIPA buffer. Protein lysate was subjected to immunoblotting with anti-phospho-PERK, anti-total-PERK, anti-phospho-eIF2α and anti-CHOP antibodies. Anti-β–actin served as a loading control. **(E,F)** The expression ratio of phospho-PERK/PERK **(E)** and elF2α/β-actin **(F)** were quantified.

To further elucidate the role of miR-200c during AD development, we examined the expression of both miR-200c and PTEN in mouse cortexes at different developmental stages of APP/PS1 transgenic mice. Interestingly, miR-200c level was increased at 4-month old mice and peaked at 6 months of age, then decreased to about baseline and stayed at the level from 9-month old and the rest of tested ages in APP/PS1 mice when compared with age-matched wild-type mice (Figure 5A). The expression of PTEN showed a reciprocal pattern to that of miR-200c (Figures 5B,C). Previous studies showed that the ER stress is induced early in AD (Endres and Reinhardt, 2013). We then examined the levels of ER stress markers including phosphorylated PERK, phosphorylated eIF2α, and CHOP (Figure 5D). The expression ratio of phospho-PERK/PERK or elF2α/β-actin were quantified in Figures 5E,F, respectively. Both phospho-PERK and phospho-eIF2α increased in APP/PS1 transgenic mice at early stages, which is similar to the expression pattern of miR-200c. CHOP is one of the ER stress markers and is upregulated at very late stages. In APP/PS1 mice brains, CHOP also appeared earlier than that in wild type mice.

### ER stress pathways are essential for Aβ–induced miR-200c expression changes

**Figure 6:**
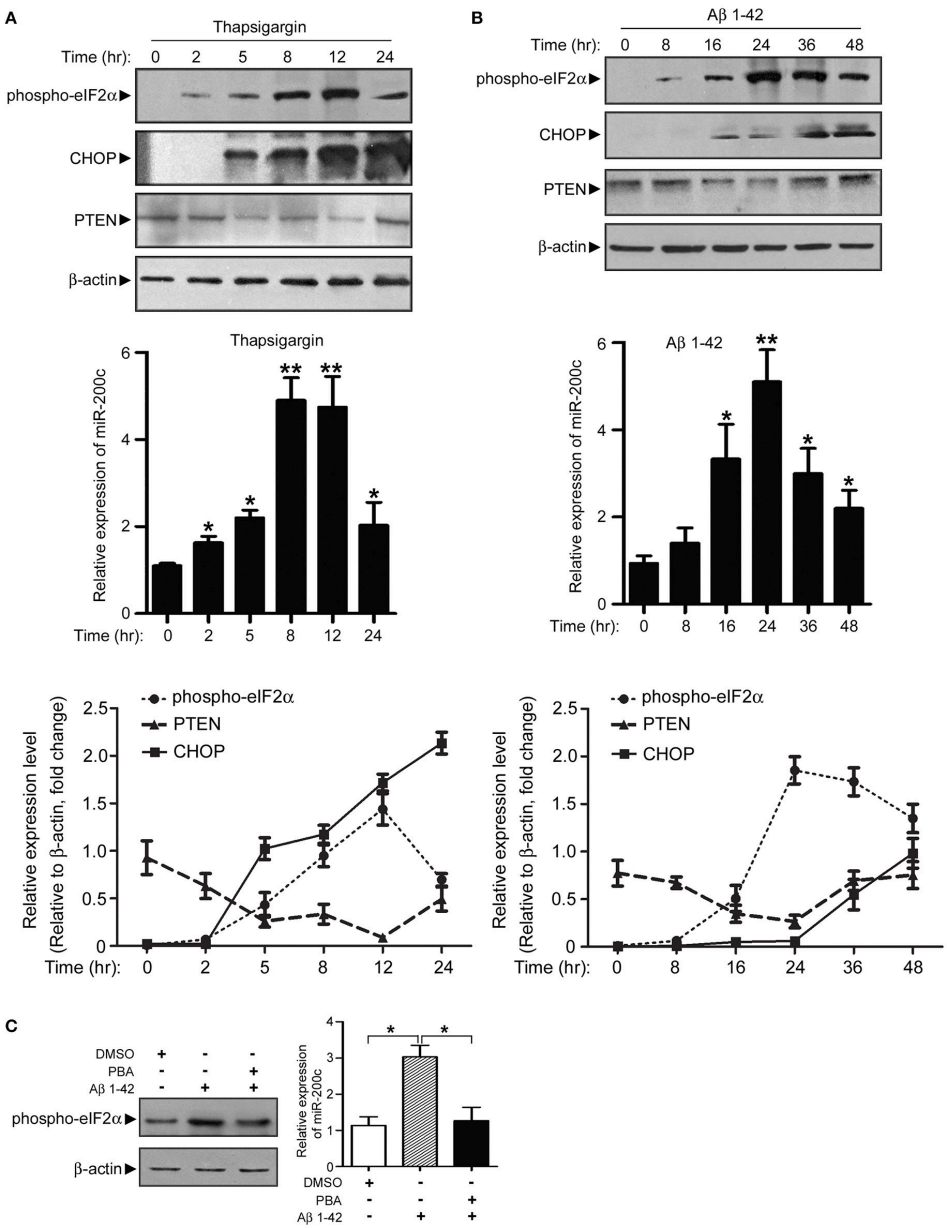
**Expression of miR-200c is regulated by Aβ-induced ER stress. (A)** PC12 cells were treated with thapsigargin and cell lysate was harvested at different time points as indicated. Phospho-elF2α, CHOP, and PTEN were detected by immunoblotting (left panel). Total RNA was also extracted and subjected to qPCR analysis of miR-200c (right panel). Expression level of miR-200c is represented as the fold change compared to time point 0. **(B)** PC12 cells were treated with Aβ, followed by similar experiments described in **(A)**. Data are represented as mean ± SEM. (^*^P < 0.05, ^**^P < 0.01, compared to time point 0). **(C)** NGF-differentiated PC12 cells were pretreated by ER stress inhibitor PBA for 2 h before 24 h Aβ treatment Phospho-eIF2α (left panel) or miR-200c (right panel) were detected by immunoblotting and qPCR, respectively. Data are represented as mean ± SEM. (^*^P < 0.01).

The correlated expression pattern of miR-200c and early ER stress markers suggested a link between Aβ-induced ER stress and microRNA expression. We found that miR-200c does not induce ER stress (Figure S3). It is plausible that the upregulation of miR-200c induced by Aβ may be mediated by ER stress. To test this hypothesis, PC12 cells were treated with the ER stress activator thapsigargin or Aβ for different time periods to induce ER stress. Increase in phospho-eIF2α and CHOP after treatment of thapsigargin or Aβ marked the induction of ER stress in cells (Figures 6A,B). MiR-200c expression increased gradually along with phosphorylated eIF2α (Figures 6A,B, middle and lower panels). Meanwhile, the level of PTEN decreased (Figures 6A,B). These results indicate that ER stress pathways are the mediators of Aβ–induced miR-200c expression. Consistent with this notion, pretreatment of neuronal cells with PBA, an ER stress inhibitor, decreased the level of phospho-eIF2α and inhibited the induction of miR-200c (Figure 6C). Taken together, our data show that ER stress pathways play an essential role in Aβ-induced miR-200c expression.

### miR-200c is up-regulated in the plasma of moderate to severe AD patients

**Figure 7:**
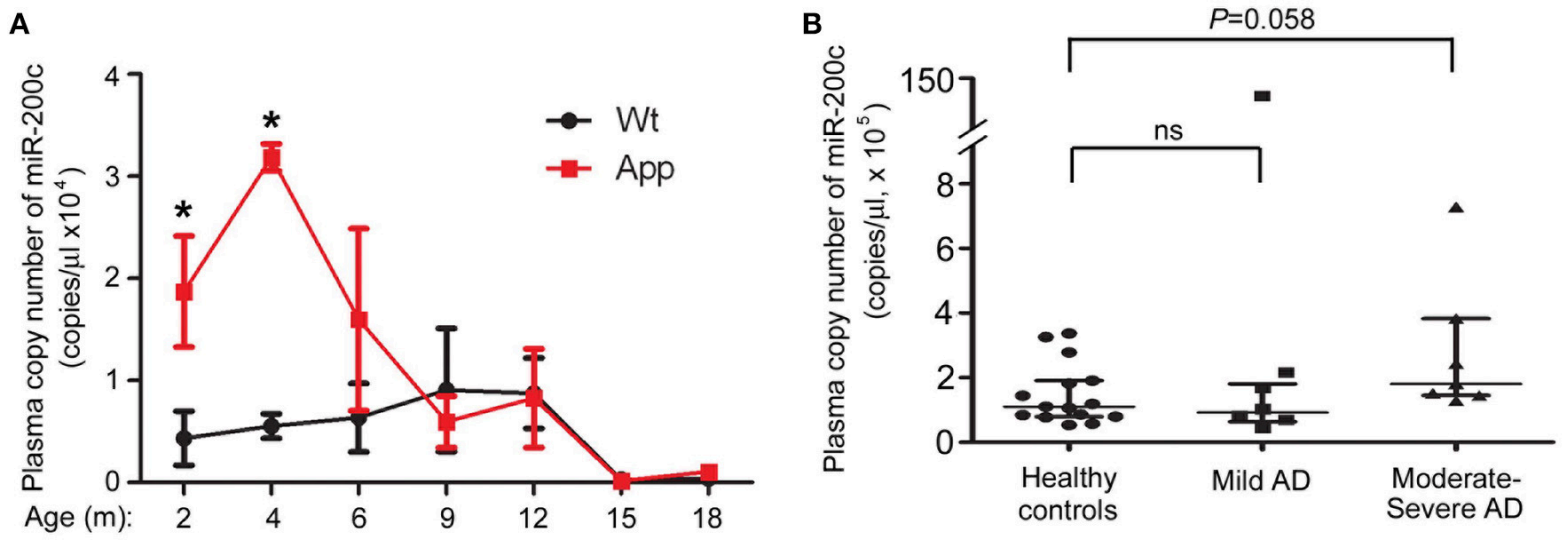
**Detection of plasma-circulating miR-200c in AD mice model and patients. (A)** Plasma samples were collected from six APP/PS1 and six WT mice at indicated ages **(A)** or from 14 AD patients and 15 age-matched healthy controls **(B)**. Absolute copies of miR-200c in plasma were examined by TaqMan qRT-PCR (^*^P < 0.05, compared to relative WT control). AD patients were divided into two groups according to the disease severity. Data are represented as mean ± SEM. Statistical significance was determined by one-way ANOVA followed by *post-hoc t*-test.

To test if the expression level of miR-200c in the peripheral blood has an indicative role for AD development, we examined plasma miR-200c level in both APP/PS1 mice and AD patients. Plasma miR-200c levels were much higher in APP/PS1 mice than wild type mice from 2 to 6 months of age (Figure 7A). After 9 months age, there were no significant differences between these two types of mice (Figure 7A). To further confirm whether plasma miR-200c in human samples is indicative for AD development, we collected 27 human plasma samples. In the 14 AD human patients (among them 7 with mild AD and 7 with moderate to severe AD), plasma miR-200c levels (range = 4.5 × 10^4^ to 7.28 × 10^5^ copies/μl plasma; mean = 2.04 × 10^5^ ± 0.50 × 10^5^ copies/μl plasma) were not significantly different from that in the healthy controls (range = 5.3 × 10^4^ to 3.36 × 10^5^ copies/μl plasma; mean = 1.49 × 10^5^ ± 0.25 × 10^5^ copies/μl plasma). When we divided the AD patients into two groups according to the disease severity, we found the plasma miR-200c levels of the 7 moderate to severe AD patients were modestly higher (Range = 1.45 × 10^5^ to 7.28 × 10^5^ copies/μl plasma, mean = 2.80 × 10^5^ ± 0.82 × 10^5^ copies/μl plasma) than that of the healthy controls (*P* = 0.058) (Figure 7B), indicating that plasma miR-200c may also be related to AD development.

## Discussion

ER stress is triggered by the accumulation of misfolded proteins within the ER lumen. Three ER transmembrane receptors IRE1α, PERK, and ATF6 are activated by the stimuli of unfolded proteins accumulation. Signaling pathways initiated by these ER stress receptors are referred to as the unfolded protein response (UPR). The function of UPR is to reduce unfolded protein levels and restore ER homeostasis (Logue et al., 2013). ER stress has been implicated in the pathogenesis of neurodegenerative diseases such as Alzheimer's disease (Endres and Reinhardt, 2013). Activated UPR signaling was thought to be an early event during Alzheimer's disease progression and was found in brains of both AD patients and AD animal models (Hoozemans et al., 2012; Endres and Reinhardt, 2013). In our study, we found Aβ peptide treatment induces phosphorylation of PERK and eIF2 α and upregulates miR-200c expression, which were also observed in APP/PS1 mouse brains (4–6 months old). MiR-200c plays protective roles in neuron proliferation and survival. Under the stress of Aβ peptide, UPR is activated and miR-200c is upregulated under an thus far unknown mechanism We hypothesized that it might be due to the activation of transcriptional factors such as XBP1, ATF4, and ATF6. The UPR signalings contributes to neuron homeostasis at the early stage of Aβ impairment at least partially via miR-200c and its targets. If the UPR is insufficient to deal with chronic exposure to Aβ stimuli, a switch to apoptotic signaling such as CHOP starts. At this late stage of ER stress induced by Aβ, miR-200c is decreased and PTEN is restored to accelerate the detrimental neuron death. So we hypothesized that in this APP/PS1 mouse model brain, at the early stages of Aβ stress (4–6 months old), UPR is activated and miR-200c increases to play the protective roles. However, when Aβ is accumulated to a threshold, the apoptosis program is started and miR-200c is decreased, which is consistent with the time scale of neuronal loss in this APP/PS1 mouse model.

Recently, miRs have been found to play important roles in ER stress process. MiRs can either regulate ER stress (Dai et al., 2010; Yang et al., 2011) or be regulated by ER stress (Groenendyk et al., 2014; Ye et al., 2015; Nolan et al., 2016). In this study, miR-200c helps to maintain the balance between survival and death during Aβ-induced ER stress. In APP/PS1 mice, plasma-circulating miR-200c is significantly elevated compared to that in WT mice at early stages (2–4 months). However, in human, only patients with moderate to severe AD have a relatively higher plasma miR-200c level, but not mild AD patients. This difference may due to the following reasons: (1) The sample size in the current human study is too small and we need more data to confirm it. (2) APP/PS1 mice are an Aβ deposition model which have much higher Aβ expression. However, AD patients have much more complicated pathogenic causes and ER stress is not very significant in mild AD. It will be valuable to examine the level of plasma miR-200c in familial AD patients that harbor APP or PS1 mutations.

PTEN serves as both tyrosine and lipid phosphatases. The enzyme is well established as a tumor suppressor (Sulis and Parsons, 2003). In the past decade, PTEN was also related to neurodegeneration. Downregulation of PTEN represses the hyperphosphorylation of tau induced by OA in SH-SY5Y cells and promotes cell survival (Chen et al., 2012). MiR-26a promotes neurite outgrowth by inhibiting PTEN expression (Li and Sun, 2013). PTEN deficiency in the brain causes defects in synaptic structure, transmission and plasticity in the hippocampus (Fraser et al., 2008). Transcription factor EB targets PTEN and reduces phospho-tau, neurofibrillary tangle pathologies and rescues neurodegeneration in rTg4510 mice (Polito et al., 2014). So far, we know that PTEN is involved in the regulation of neurogenesis, neurite outgrowth, synaptogenesis and synaptic plasticity (Zhou and Parada, 2012). Importantly, the PDZ-binding domain of PTEN plays the key role in AD-associated post-synaptic dysfunctions (Knafo et al., 2016). In our study, we found PTEN is inhibited at early stage under the Aβ stress in both cell and animal models, indicating that PTEN is related to not only neurofibrillary tangle pathologies but also Aβ toxicity. Consistent with this notion, we found that overexpression of miR-200c and knocking down of PTEN protect neurite outgrowth in both PC12 cells and SCG neurons. However, such phenomena were not observed in cultured primary cortical neurons. As PTEN was found to inhibit TrkA expression and NGF signaling in PC12 cells (Musatov et al., 2004), whereas most cortical neurons express TrkB instead, we hypothesized that Aβ-induced PTEN expression reduction only played protective roles in TrkA-positive cells. In the cortex, about 20% of the neurons which are cholinergic neurons express TrkA and depend on NGF signaling for their survival and excitability (Rattray, 2001). Consistently, in AchE-positive cortical neurons, overexpression of miR-200c or inhibition of PTEN promoted neurite outgrowth. One important characteristic of AD pathology is the loss of cholinergic neurons that may contribute to memory lost (Jürgensen and Ferreira, 2010). Our data indicate that Aβ-miR-200c-PTEN pathway may play important roles in cholinergic neurons at the early stage of AD.

In conclusion, our results show that at the early stage of Aβ damage, ER stress induces upregulation of miR-200c to inhibit PTEN expression and protect neurons from Aβ toxicity. This process mainly happens in the cholinergic neurons. Chronic exposure to Aβ peptide disrupts homeostasis of neurons and results in the face of miR-200c response and neuronal cell death.

## Author contributions

QW carried out the molecular and cellular studies, participated in the animal experiments and drafted the manuscript. XY carried out the immunostaining assays and revised the manuscript. YX participated in the animal experiments including tissue collection and RNA/protein extraction and helped to revise the manuscript. HZ participated in human sample collection, performed the circulating miRNA detection and revised the manuscript. JM participated in collecting the human samples, performed the circulating miRNA detection and drafted the manuscript. WZ participated in the design of the study, performed the statistical analysis and helped to revise the manuscript. JW conceived of the study, and participated in its design and coordination and helped to draft the manuscript. All authors read and approved the final manuscript.

## Funding

This work was supported by National Basic Research Program of China (973 Program) Grant 2014CB910204, the National Key Research and Development Program (2016YFA0501900), National Natural Scientific Foundation of China (Grant No. 81300922, 81371737, and 81571043), Natural Science Foundation of Guangdong Province (2016A030312016) and Shenzhen Basic Research Grants (Grant No. JCYJ20140416144209745 and JCYJ20160229153100269).

### Conflict of interest statement

The authors declare that the research was conducted in the absence of any commercial or financial relationships that could be construed as a potential conflict of interest.

## Supplementary material

Figure S1 **miR-200 inhibitors up-regulate PTEN expression**. HCT-116 cells were transfected with different combinations of miR-200 inhibitors as indicated. PTEN expression was detected by Western blotting, with GAPDH serving as a loading control. The experiments were repeated for three times. Gray degree values are quantified by Image J software. The result was shown as the mean ± SEM. [Lower panel, ^*^*P* < 0.05, compared to mimic control].

Figure S2 **miR-200c is important for neurite outgrowth in cultural SCG neuron. (A–C)** SCG neurons were transfected with miR-200c mimic or inhibitor together with GFP at DIV0. After transfection, cells were cultured for 72 h. Neurite outgrowth was measured as described in Figure 3. **(D–F)** PTEN was overexpressed or knocked-down by the transfection of GFP-PTEN or PTEN siRNA, followed by measurement on neurite growth. Data are represented as mean ± SEM. (^*^*P* < 0.05. Scale bars: 20 μm). **(G)** Rat cortical neurons at DIV0 were transfected with miR-200c mimic or inhibitor with GFP. Cells were cultured for 3 days. Total or the longest neurite length of cortical neurons were measured as described above. Three independent experiments were performed. Data are represented as mean ± SEM. **(H)** After the transfection the same as in **(G)**, AChE positive cells was visualized by immunostaining. Total or the longest neurite length of AChE positive cells were measured as described above. Three independent experiments were performed. Data are represented as mean ± SEM. (^**^*P* < 0.01).

Figure S3 **miR-200c cannot induce ER stress**. PC12 cells were transfected with miR-200c mimic or inhibitor followed by NGF treatment for 48 h and cell lysate was harvested. Protein lysate was subjected to immunoblotting with anti-phospho-eIF2α, anti-phospho-PERK, anti-CHOP, and anti-total-PERK antibodies. Anti-GAPDH served as a loading control.

## Abbreviations

AD: Alzheimer's disease
Aβ: beta amyloid peptide
DIV: differentiation *in vitro*
ER: endoplasmic reticulum
MiR: MicroRNA
NFT: Intracellular neurofibrillary tangles
PTEN: phosphatase and tensin homolog deleted on chromosome ten
SCG: Superior cervical ganglia
UPR: unfolded protein response.
