# Supplementary figures and images for: The Protective Role of microRNA-200c in Alzheimer's Disease Pathologies Is Induced by Beta Amyloid-Triggered Endoplasmic Reticulum Stress
Source: Front Mol Neurosci. 2016 Dec 8;9:140. doi: 10.3389/fnmol.2016.00140 (PMC5143617; doi:10.3389/fnmol.2016.00140)

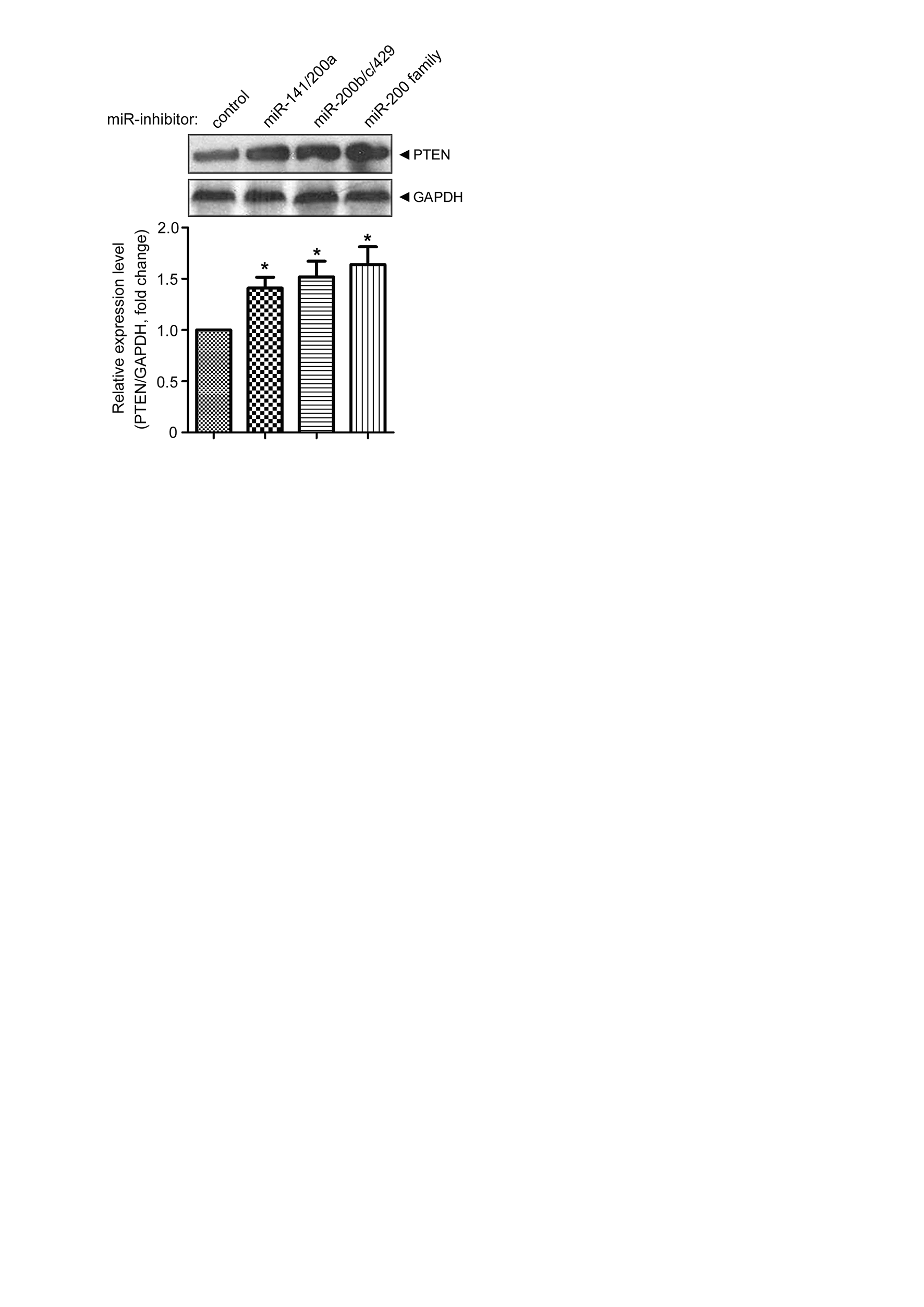

Supplement: Figure S1 — miR-200 inhibitors up-regulate PTEN expression. HCT-116 cells were transfected with different combinations of miR-200 inhibitors as indicated. PTEN expression was detected by Western blotting, with GAPDH serving as a loading control. The experiments were repeated for three times. Gray degree values are quantified by Image J software. The result was shown as the mean ± SEM. [Lower panel, *P < 0.05, compared to mimic control]. [file Image1.TIF]

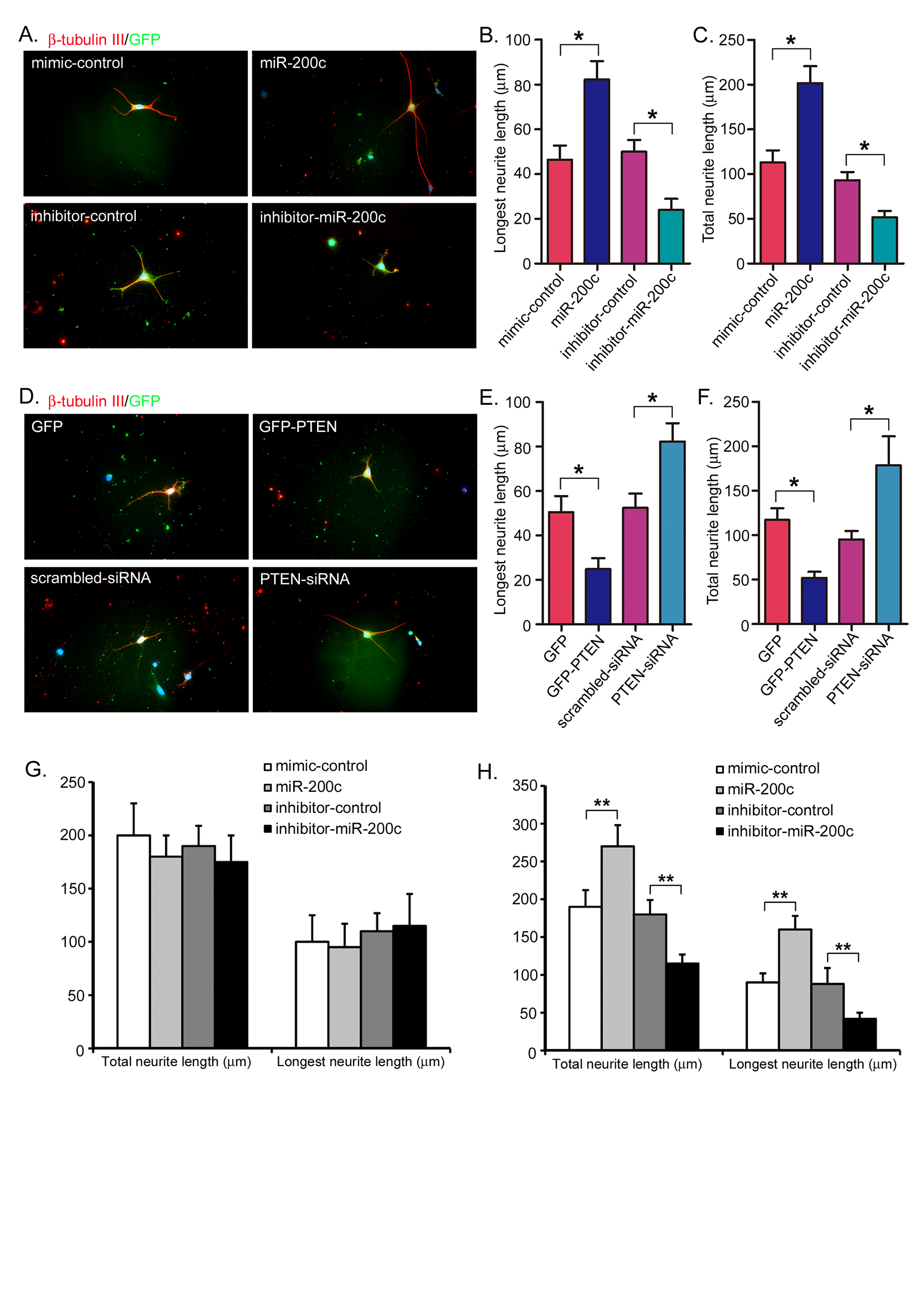

Supplement: Figure S2 — miR-200c is important for neurite outgrowth in cultural SCG neuron. (A–C) SCG neurons were transfected with miR-200c mimic or inhibitor together with GFP at DIV0. After transfection, cells were cultured for 72 h. Neurite outgrowth was measured as described in Figure 3. (D–F) PTEN was overexpressed or knocked-down by the transfection of GFP-PTEN or PTEN siRNA, followed by measurement on neurite growth. Data are represented as mean ± SEM. (*P < 0.05. Scale bars: 20 μm). (G) Rat cortical neurons at DIV0 were transfected with miR-200c mimic or inhibitor with GFP. Cells were cultured for 3 days. Total or the longest neurite length of cortical neurons were measured as described above. Three independent experiments were performed. Data are represented as mean ± SEM. (H) After the transfection the same as in (G), AChE positive cells was visualized by immunostaining. Total or the longest neurite length of AChE positive cells were measured as described above. Three independent experiments were performed. Data are represented as mean ± SEM. (**P < 0.01). [file Image2.TIF]

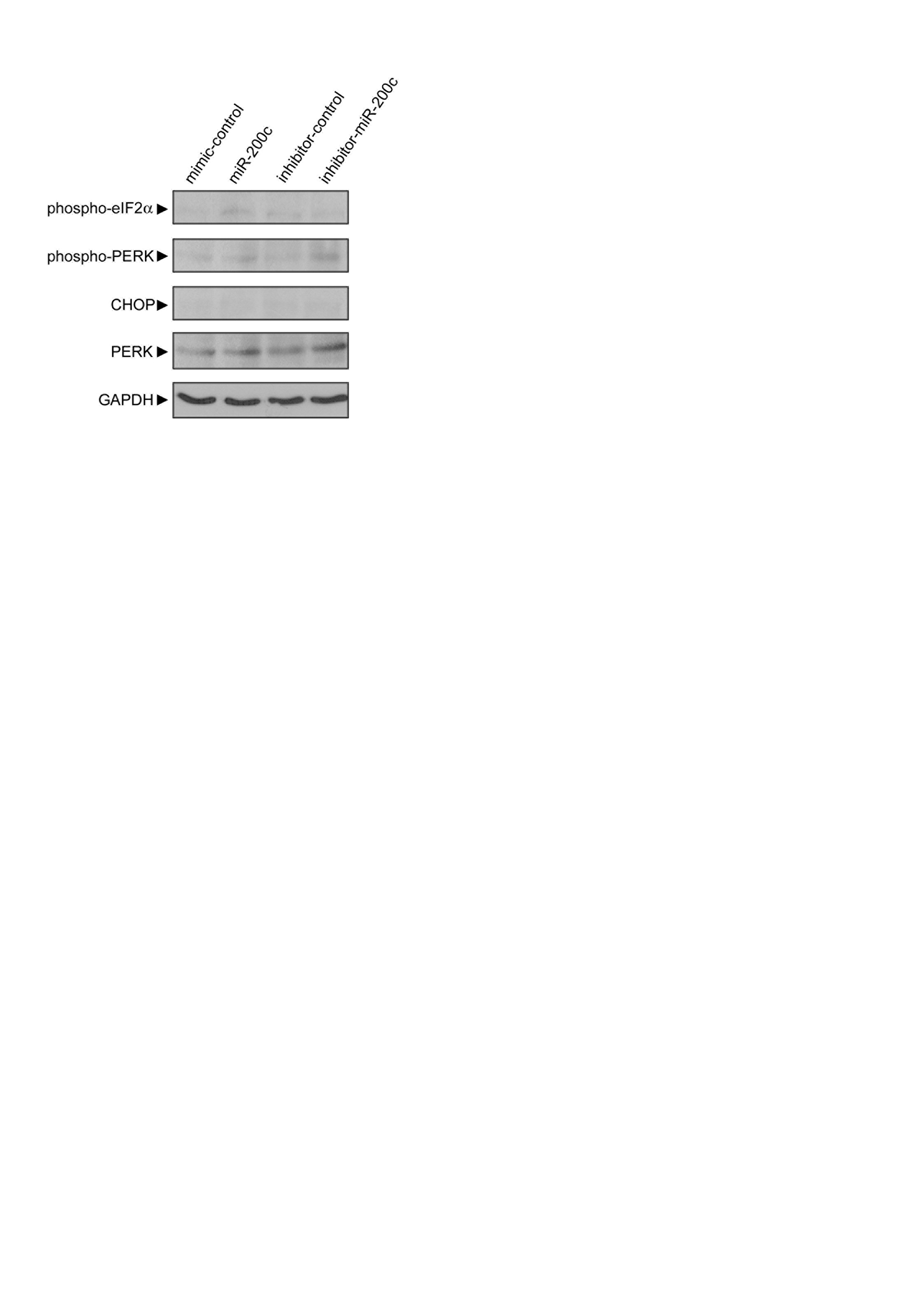

Supplement: Figure S3 — miR-200c cannot induce ER stress. PC12 cells were transfected with miR-200c mimic or inhibitor followed by NGF treatment for 48 h and cell lysate was harvested. Protein lysate was subjected to immunoblotting with anti-phospho-eIF2α, anti-phospho-PERK, anti-CHOP, and anti-total-PERK antibodies. Anti-GAPDH served as a loading control. [file Image3.TIF]
